# Can composite packaging and selective enamel etching affect the clinical behavior of bulk-fill composite resin in posterior restorations? 24-month results of a randomized clinical trial

**DOI:** 10.1590/1678-7757-2022-0323

**Published:** 2023-01-27

**Authors:** Marcos de Oliveira BARCELEIRO, Chane TARDEM, Elisa Gomes ALBUQUERQUE, Leticia de Souza LOPES, Stella Soares MARINS, Luiz Augusto POUBEL, Roberta BARCELOS, Romina ÑAUPARI-VILLASANTE, Alessandro Dourado LOGUERCIO, Fernanda Signorelli CALAZANS

**Affiliations:** 1 Universidade Federal Fluminense Departamento de Odontologia Restauradora Nova Friburgo Rio de Janeiro Brasil Universidade Federal Fluminense, Departamento de Odontologia Restauradora, Nova Friburgo, Rio de Janeiro, Brasil.; 2 Universidade Federal Fluminense Departamento de Odontologia Restauradora Niterói Rio de Janeiro Brasil Universidade Federal Fluminense, Departamento de Odontologia Restauradora, Niterói, Rio de Janeiro, Brasil.; 3 Universidade Federal de Juiz de Fora Departamento de Odontologia Restauradora Juiz de Fora, Minas Gerais Brasil Universidade Federal de Juiz de Fora, Departamento de Odontologia Restauradora, Juiz de Fora, Minas Gerais, Brasil.; 4 Universidade Estadual de Rio de Janeiro Departamento de Odontologia Restauradora Rio de Janeiro Rio de Janeiro Brasil Universidade Estadual de Rio de Janeiro, Departamento de Odontologia Restauradora, Rio de Janeiro, Rio de Janeiro, Brasil.; 5 Universidade Estadual de Ponta Grossa Departamento de Odontologia Restauradora Ponta Grossa Paraná Brasil Universidade Estadual de Ponta Grossa, Departamento de Odontologia Restauradora, Ponta Grossa, Paraná, Brasil.

**Keywords:** Composite resin, Dental adhesives, Clinical trial, Dental restoration failure

## Abstract

**Objectives:**

This is a double-blind, split-mouth, randomized clinical study that aims to evaluate the influence of bulk-fill composite packaging presented in syringes (BSy) and capsules (BCa), and the effect of selective enamel etching (SEE) on the clinical performance of class I and II bulk-fill resin composite restorations after 24 months.

**Methodology:**

A total of 295 class I or class II restorations were performed on 70 patients. One universal adhesive was applied in all restorations. SEE was used in 148 restorations and self-etching mode (SET) in 147 restorations. After the adhesive application, cavities were restored with Filtek Bulk-fill Posterior Restorative in syringes (BSy), Filtek One Bulk-fill in capsules (BCa), or Filtek Supreme Ultra in syringes with the incremental technique (In). All restorations were evaluated using the FDI criteria after one week and after six, 12, and 24 months. Kaplan-Meier survival analysis and Pearson’s Chi-square test were used (α=0.05) for statistical analysis.

**Results:**

After 24 months, 62 patients were evaluated and four restorations were lost due to fracture (one for SEEBSy, two for SEEIn, and one for SETIn). No significant differences in the fracture and retention rate were found between groups (p>0.05). SEE showed significantly fewer marginal adaptation defects than SET (p<0.05). BCa and BSy groups showed fewer marginal discrepancies compared to In (p<0.05). Restorations performed with BCa showed less color mismatch than BSy or In (p<0.05).

**Conclusion:**

Although all restorations exhibited satisfactory clinical performance after 24 months of clinical service, the clinical behavior of class I and II restorations’ improved when performed with a bulk-fill composite in capsules, mainly when associated with a universal adhesive applied with SEE.

## Introduction

For many years, the incremental technique has been the most suitable method for restoring teeth using composite resin, with increments of up to 2 mm in thickness. This technique facilitates a proper light curing of the composite and reduces the stress generated by polymerization shrinkage.^1^ However, the incremental technique has some disadvantages, such as the possibility of internal flexure on the preparation of the tooth walls due to each layer of cured composite, the requirement of good adaptation and bonding between increments, the possibility of voids forming between each increment, and prolonged clinical time.^2^

To minimize these problems and simplify the restorative technique, bulk-fill composite resins have been introduced to the market. Modifications to this material allow for the use of increments of 4-5 mm in thickness, without negatively affecting the light curing of the deeper layers. Due to the low polymerization shrinkage, it is possible to insert increments joining more than two walls, unlike with conventional composite resins.^3^ Studies suggest that these materials reduce the possibility of cusp deflection, the incorporation of voids, material contamination,^4^ and clinical care time.^5^

The first bulk-fill composite resins introduced to the market were flowable bulk-fill or low-viscosity bulk composite resins, whose effectiveness has been evaluated and approved in long-term studies.^6 , 7^ Regular bulk-fill resins that were later introduced to the market still lack further scientific evidence, as few studies have been conducted to evaluate occlusal or occlusal-proximal restorations performed with bulk-fill resins with 24 or more months of clinical follow-up.^8 - 13^

On the other hand, several commercial composite resins were recently launched in single doses (capsules) in addition to the traditional presentation in syringes. According to the manufacturers, this new presentation seems to help clinicians with infection control during restorative procedures, apart from being easily handled with a dispenser and being less time-consuming because it does not require using a spatula to pick a portion of the material from the syringe, which is necessary in some cases. However, a recently published study^5^ showed no significant difference in application time when using two bulk-fill composites presented either in capsules or in syringes. To our knowledge, no clinical studies have been conducted to determine if the insertion and packaging of the composite resin (syringe vs. capsules) affect the clinical behavior and longevity of posterior restorations.

Universal adhesive systems are another group of materials that have been developed aiming to facilitate and speed up the performance of adhesive restorative procedures due to their versatility.^14^ These materials have been extensively evaluated in laboratory and clinical research, usually in non-carious cervical lesions, and its results seem very promising.^15 - 17^ However, only a few medium-term evaluations were performed on class I or II occlusal restorations using these universal adhesive systems combined with bulk-fill composites,^12 , 18 , 19^ and none of them have evaluated the effect of selective enamel etching (SEE) associated with universal adhesives.

Therefore, this randomized clinical trial aims to evaluate, after 24 months of clinical evaluation, the clinical performance of class I and class II restorations performed with universal adhesives applied either in the SEE or in the self-etch (SET) mode and to determine if bulk-fill composite resins packaged either in syringes or in capsules are reliable substitutes for composite resins inserted using the incremental technique.

The null hypotheses tested were: i) there is no significant difference in the fracture and retention rates of class I and class II restorations made with different composite packaging (syringe vs. capsule) and the incremental technique when evaluated using FDI criteria, ii) there is no statistical difference in the fracture and retention rates of class I and class II bulk-fill restorations made with SEE compared to SET adhesive strategy when evaluated using FDI criteria, iii) there is no statistical difference in the secondary outcomes (recurrence of caries, anatomic form, proximal contact quality, color match, marginal staining, marginal adaptation, postoperative sensitivity, and patient perception) of class I and class II bulk-fill restorations made with different composite packaging (syringe vs. capsule) compared to the incremental technique when evaluated using FDI criteria, and iv) there is no statistical difference in the secondary outcomes of class I and class II bulk-fill restorations made with the SEE compared to SET adhesive strategy when evaluated using FDI criteria.

## Methodology

### Ethics approval and protocol registration

The Ethics Committee of the School of Fluminense Federal University, Nova Friburgo, RJ, Brazil - acrescentar após School of Dentistry reviewed and approved this study under protocol number 2.063.508. This study was registered in ClinicalTrial.gov (NCT03343184) and was conducted and reported in accordance with the CONSORT statement.^20^ All participants were informed about the objectives and nature of the study and signed an informed consent form before their inclusion in the study.

### Trial design, settings, and location of data collection

This was a double-blind (patient and examiner), split-mouth, prospective, and randomized clinical trial. This study was conducted at the clinics of the School of Fluminense Federal University, Nova Friburgo, RJ, Brazil, from August 17, 2017, to September 30, 2017. The 24-month follow-up evaluation was conducted from August, 2019 to September, 2019.

### Participants recruitment

Patients were recruited in the order in which they appeared for screening sessions in the Fluminense Federal University dental clinics, thus forming a sample of convenience. Two calibrated dental residents (C.T. and F.C.) recruited the patients. The calibration was performed before the start of the screening sessions and teeth selection. In two consecutive days, the investigators clinically and radiographically evaluated 10 teeth with class I and II lesions. After the evaluation, inter-examiner and intra-examiner agreements were estimated, and a score of at least 85% was required for dentists to be considered calibrated.^21^

### Eligibility criteria

Participants had to be in good general health, be at least 18 years, have an acceptable oral hygiene level according to the Simplified Oral Hygiene Index (OHI-S),^22^ have at least three posterior teeth with a carious lesion that required restoration or deficient posterior restoration in need of replacement and repair that is not feasible due to deep caries or exposed dentin not accessible for repair, generalized major gaps or irregularities, partial or complete loss of restoration, and/or multiple fractures (more than half of the restoration), that cause several adverse effects, including pain.^23^

Patients with severe or chronic periodontitis (teeth with probing pocket depth more than 4 mm with bleeding on probing and clinical attachment loss more than 3 mm in more than four teeth^24^ were excluded. Participants with known allergies to resin-based materials or any other material used in this study, pregnant or breastfeeding women, patients using anti-inflammatories, analgesics, or psychotropic drugs within 15 days of the restorative procedure were also excluded.

### Characteristics of the teeth/cavities to be included

The teeth intended for restoration had to be in occlusion with their natural antagonist tooth and adjacent teeth. To identify occlusal interferences in the intercuspal position and in the lateral movements, articulating paper was used. If the tooth presented an occlusal interference, an occlusal adjustment was performed. Teeth requiring endodontic treatment (evaluated by radiography and by the cold pulpal sensitivity test [Roeko-Endo-Frost, Coltène / Whaledent, Langenau, Germany]) were excluded.

The dental cavities had to be class I or class II (involving the occlusal surface) of a depth greater than 2 mm, evaluated by means of a bitewing radiograph and ruler in vital teeth. Following the American Dental Association Caries Classification System (ADA CCS), the extension of the included carious cavities had to be moderate (enamel breakdown with non-cavitated carious dentin) or advanced (full cavitation through the enamel and dentin clinically exposed). Radiographically, for class II lesions, the radiolucency had to be extended to the outer one-third of dentin, into the middle one-third of dentin, or into the inner one-third of dentin.^25^

### Sample size calculation

The average of annual failure rate of bulk-fill composite resin restorations in randomized clinical trials was 2.5%.^6 , 26^ Therefore, the overall success rate of bulk-fill composite restorations would be approximately 95% after two years of clinical service. With an α of 0.05, a power of 90%, and a two-sided test, the minimal sample size was 44 restorations in each group to detect a difference of 25% between groups. However, considering the risk of patient losses intrinsic to randomized clinical trials, we chose to increase the number of restorations in each group by 20%. Thus, 53 cavities per group were included. These calculations were performed at www.sealedenvelope.com for one of the researchers (A.L.).

### Randomization, allocation, and blinding

**Figure 1:**
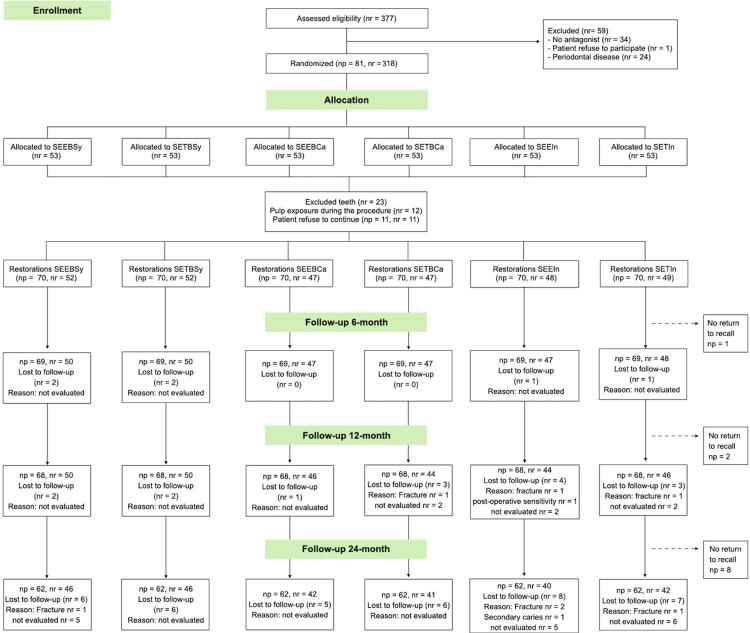
Participant flow diagram in the different phases of the study design. Abbreviations: np – number of participants; nr – number of restorations; (*) SEEBSy, self-etch with selective enamel etching and bulk in syringe; SETBSy, self-etch and bulk in syringe; SEEBCa, self-etch with selective enamel etching and bulk in capsule; SETBCa, self-etch and bulk in capsule; SEEIn, self-etch with selective enamel etching and incremental composite; SETIn, self-etch and incremental composite

Initially, 377 teeth were evaluated, but 59 were excluded because of the reasons described in Figure 1 . After the screening sessions, 81 patients were selected and scheduled to perform the restorations. A total of 318 teeth, treated as research subjects, were randomized in blocks of six to ensure an equal number of restorations in each of them. The randomization process was performed using a free program available at http://www.sealedenvelope.com by a researcher uninvolved in any experimental phases (R.Ñ.).

The randomization list was numbered consecutively and was individually placed in opaque sealed envelopes opened on the day of the restorative intervention to prevent disclosure of the randomization scheme. The operator who conducted the interventions was not blinded. However, participants and examiners were kept blind to the group allocation during examinations.

### Baseline characteristics of the selected teeth

The characteristics of each patient (gender, age) and the teeth intended for restoration (tooth type, arch, cavity type, presence of spontaneous sensitivity before restoration, number of restored surfaces, and restoration reason) were evaluated and recorded by four trained and calibrated operators before the placement of the restorations. These operators were also involved in the selection of participants and conduct of the restorative procedures ( Table 1 ).

**Table 1:** Characteristics of arches and cavities

Variable	Group (n)						
	SEEBSy	SETBSy	SEEBCa	SETBCa	SEEIn	SETIn	Total
	(n=52)	(n=52)	(n=47)	(n=47)	(n=48)	(n=49)	(n=295)
**Tooth**							
Premolar	19	24	19	30	14	20	126
Molar	33	28	28	17	34	29	169
**Cavity classification**							
Class I	31	32	24	22	35	32	176
Class II – 1 proximal surface	16	13	15	20	10	15	89
Class II – 2 proximal surfaces	5	7	8	5	3	2	30
**Restoration depth**							
2–3.9mm	31	26	24	30	27	26	164
≥4mm	21	26	23	17	21	23	131
**Caries**							
Absent	39	41	26	34	42	43	225
Present	13	11	21	13	6	6	70
**Restoration reason**							
Caries	13	11	21	13	6	6	70
Restoration change for esthetic reasons	29	32	17	20	35	34	167
Restoration change for other reasons	10	9	9	14	7	9	58

(*) SEEBSy, self-etch with selective enamel etching and bulk in syringe; SETBSy, self-etch and bulk in syringe; SEEBCa, self-etch with selective enamel etching and bulk in capsule; SETBCa, self-etch and bulk in capsule; SEEIn, self-etch with selective enamel etching and incremental composite; SETIn, self-etch and incremental composite.

The four operators (C.T., E.A., L.L., and S.M.) were dentists with more than five years of clinical experience in Operative Dentistry and were calibrated by the study director (M.B.) to perform all restorative procedures. For the calibration, the study director placed one restoration of each group to familiarize the operator with the steps involved in the protocol. Then, the operators placed three restorations in a clinical setting, one of each group, under the supervision of the study director. Any defects of the restorative protocol were identified and discussed with the operator before starting the study. Once these procedures were completed, the operators were considered calibrated to perform the restorative procedures. The calibrated operators restored all teeth under the supervision of the study director.

### Intervention: restorative procedure

The patients received instructions for oral hygiene and dental prophylaxis of the tooth surface with pumice and water in a rubber cup, followed by rinsing and drying, with the aim of removing any remaining dental plaque. The proper shade of the resin composite was determined using a shade guide (Vita Classical, Vita Zahnfabrik, Bad Säckingen, Germany) before the restorative procedures. Local anesthesia was applied with a 3% mepivacaine solution (Mepisv 3%, NovaDFL, Rio de Janeiro, Brazil) and rubber dam isolation was performed. The cavity design was defined by the extension of carious tissue or removal of the defective restoration, and it was performed as conservatively as possible. It did not involve any cusps and all gingival margins had sound enamel.

The defective restorations were removed using a spherical diamond bur (# 1013; KG Sorensen, Barueri, Brazil) mounted in a high-speed handpiece with air-water spray. After removing the defective restorations, in the case of primary caries or the necessity of caries removal, after removing the failure restorations, the criteria used for the removal of carious tissue was selective, maintaining the affected dentin layer. This procedure was performed using hand instruments and slow-speed tungsten carbide burs (# 2 and 4; KG Sorensen, Barueri, Brazil). Bevels were not performed in cavity walls to avoid unnecessary loss of dental tissue. After cavity preparation, the width, length, and depth of occlusal and proximal boxes were measured using a periodontal probe (# 6 Satin Steel Handle, mm, Hu-Friedy, Chicago, IL, USA) and recorded. The deepest measure was used as reference to determine the number and thickness of the increments needed to fill the cavity with the composite resins.

**Figure 2:**
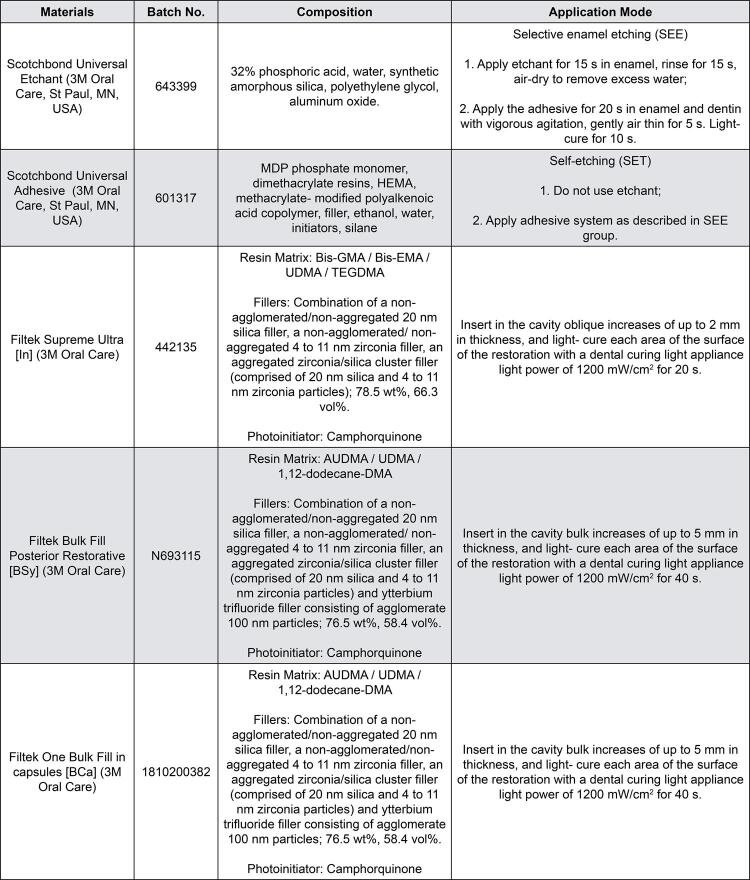
Composition and application mode of materials used in the study * MDP = 10-Methacryloyloxydecyl dihydrogen phosphate; HEMA = 2-hydroxyethylmethacrylate; Bis-GMA = bisphenol-A glycidyl methacrylate; Bis-EMA = bisphenol-A ethoxylated dimethacrylate; UDMA = urethane dimethacrylate; TEGDMA = triethyleneglycol dimethacrylate; AUDMA = aromatic urethane dimethacrylate; 1,12-dodecane-DMA = 12-dodecane dimethacrylate.

At this point, the envelopes were opened and the operators were revealed which restorative technique they would use based on the combination of composite packaging material and adhesive strategy. The Scotchbond Universal adhesive system, also known as Single Bond Universal in some countries (3M Oral Care, St Paul, MN, USA), was applied either in the SET mode with SEE or in the SET mode, following the manufacturer’s instructions ( Figure 2 ). After the adhesive application, in class II cavities, pre-contoured metal matrices (Unimatrix, TDV, Joinville, SC, Brazil) and proximal wedges were placed and adapted to obtain the proximal contour of the restoration. After that, teeth were restored with Filtek Bulk Fill Posterior Restorative composite resin (BSy) in syringes (3M Oral Care) or Filtek One Bulk Fill (BCa) in capsules (3M Oral Care) with increments of no more than 4 mm in thickness; or with the oblique incremental technique (In) using the Filtek Supreme Ultra nanofilled composite resin (3M Oral Care) with 2 mm increments in thickness.

For the In group, each increment of composite resin was removed from the compule, shaped into a ball using the right thumb and index finger, and then placed in the cavity with a resin spatula. The operators wore new and clean gloves immediately before the restorative procedure to avoid contamination with saliva or carious tissue removal. Gloves and spatulas were cleaned with 70% alcohol and dried with absorbent paper between each incremental layer. In the BSy groups, one 4-mm-thick layer was placed at the bottom of the cavity, as described in the In group. However, for BCa groups, the material was dispensed using the Mark IIIP™ Speed Slot Syringe (Centrix, Shelton, CT, USA), and the composite resin was inserted directly into the cavity without any previous manipulation. Batch numbers, compositions of materials, as well as adhesive and restorative procedures used in the study according to each group are detailed in Figure 2 .

Adhesive system and restorative materials photopolymerization were performed using the Radii Cal (SDI, Victoria, Australia) light unit at 1,200 mW/cm^2^ , which was evaluated before each restoration with a radiometer (Hilux Led Max Curing light meter, First Medica, Greensboro, NC, USA). The light unit tip was placed as close and perpendicular as possible to the occlusal surface of the teeth, as some light reduction was expected due to the depth of the cavity. After the removal of the metal matrix, the proximal regions of class II restorations were additionally polymerized on the buccal and lingual/palatal surfaces for 10 s. Once the restorative procedures were finished, the rubber dam was removed, occlusal adjustments were performed using articulating paper (Bausch, Nashua, NH, USA), and premature contacts were removed using fine and extra-fine diamond points (# 1190F and # 1190FF, KG Sorensen). Restorations were polished with spiral discs (Sof Lex spiral, 3M Oral Care) and proximal contacts were checked with dental floss and adjusted with sanding strips (3M Oral Care) if necessary. At the end of the restorative procedure, each patient was scheduled for the subsequent evaluation (1 week) and then for the next follow-ups (6-, 12-, and 24-months). Therefore, the restorations in each patient had a particular scheduled time.

### Clinical evaluation

The postoperative sensitivity was evaluated over seven days, by the patients themselves, using two scales. A numerical rating scale (NRS), with five categories of how much sensitivity each tooth had [0 (none), 1 (mild), 2 (moderate), 3 (considerable), or 4 (severe)], and a Visual Analogue Scale (VAS), a 100 mm long straight line with scores 0 (no sensitivity) and 100 (unbearable pain) at each end. The patient was instructed to mark where their postoperative sensitivity was located along this spectrum. The patient got a form for each restored tooth, with the two scales replicated seven times, and was instructed to mark the specific day of the record of sensitivity and indicate whether it was spontaneous or stimulated. In the case of stimulated sensitivity, they were asked to indicate the cause of the sensitivity (i.e., chewing, heat, cold, or another stimulus).^5^

Two experienced and calibrated examiners (L.P. and R.B.), not involved in the restorative procedures, evaluated the restorations according to different functional, esthetical, and biological properties present in the World Dental Federation criteria (FDI)^27^ after one week and after six, 12, and 24 months of the clinical service. As part of the training, the examiners observed 10 representative photographs of each score for each criterion. They evaluated 10 subjects each on two consecutive days. These subjects had class I and class II restorations and did not participate in this study. An inter-examiner and intra-examiner agreement of at least 85% was required before starting the evaluation.^21^ Each examiner used a standardized paper report form at each recall time, so they were kept blind to previous evaluations during the follow-up recalls.

The primary outcome was fracture and retention, and the secondary outcomes were marginal adaptation, proximal contact quality (for class II restorations), patient’s perception, marginal staining, color match, anatomic form, postoperative sensitivity, and recurrence of caries. The proximal contact and cervical adaptation for class II restorations were evaluated using dental floss and bitewing radiography when examiners considered it necessary. Variables were ranked following the FDI criteria categories: clinically very good, clinically good, clinically sufficient/satisfactory, clinically unsatisfactory but repairable, and clinically poor where replacement is required. Both examiners evaluated all restorations once and independently, reaching a consensus before the participant was dismissed.

All restorations scored as clinically unsatisfactory or poor by FDI criteria at one recall were accounted as a cumulative failure at the next follow-up evaluation. Each failed restoration was replaced with a new composite resin restoration.^28^ These new restorations were not included as part of the study for further evaluation. Participants’ restorations whose evaluation was not possible to perform were considered lost to follow-up.

### Statistical analysis

The statistician was blinded to the type of study groups. The statistical analysis followed the intention-to-treat protocol, in accordance with the CONSORT.^20^ Descriptive statistics were used to describe the distributions of the evaluated criteria. For statistical purposes, the FDI criteria were dichotomized into two categories: no intervention required (clinically very good, clinically good, and clinically sufficient/satisfactory) or intervention required (clinically unsatisfactory but repairable, and clinically poor where replacement is required). Missing outcome data due to missing participants were analyzed following the imputed case analysis approach, in which all missing participants in each intervention were assumed to have experienced the event (failure).^29^

For the primary outcome fracture and retention, the survival rates of the different research groups were calculated using the Kaplan–Meier, estimating the hazard ratios (HR) and 95% confidence intervals. The logrank test was used to compare the survival distributions of these restorations (α=0.05). The absolute and relative risks of all approaches were also estimated. A 95%CI was reported. Cohen’s kappa statistics were used to evaluate inter-examiner agreement (α=0.05) (MedCalc Software, Version 19.1, Ostend, Belgium). For the secondary outcomes, the restoration groups for each category were compared using the Pearson’s Chi-square test, and the Cochran Q-test was used to compare the changes across different time points within each restorative material (α=0.05) (IBM SPSS version 22.0, SPSS, Chicago, IL, USA).

## Results

A total of 318 teeth in 81 subjects (35 men and 46 women) were selected, totaling 75 participants with four restorations and six participants with three restorations. The restorations were randomized in blocks of six experimental groups (n=53). During the preparation of the cavities, 12 teeth had to be removed from the study because of pulp exposure and 11 teeth because the patient refused to continue participating in the clinical trial; thus, the final number of restorations performed was 295, distributed among the six research groups. All subjects attended the control visit after one week. One subject did not attend to recall at the six-month evaluation; two subjects, to the 12-month, and eight subjects to the 24-month ( Figure 1 ).

**Figure 3:**
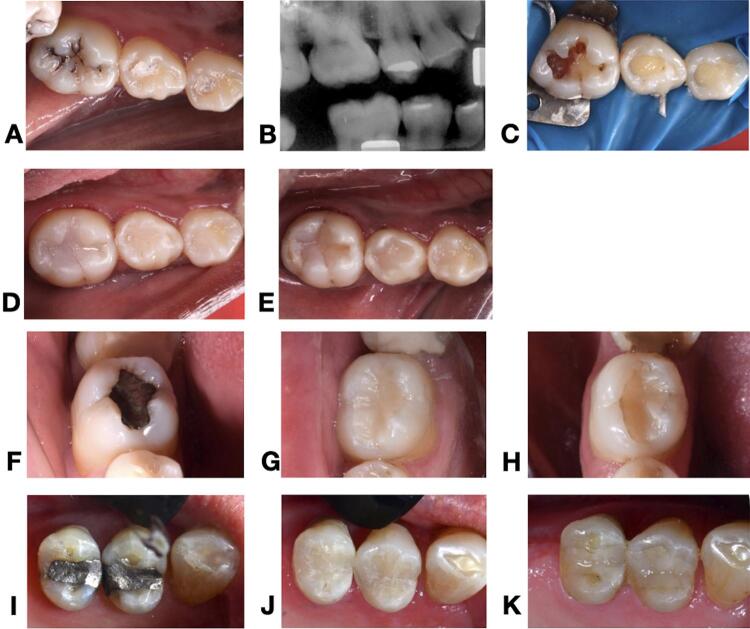
Representative photographs of clinical procedure and follow-up evaluation. (A) Baseline clinical photography of teeth 44, 45, and 46, (B) Baseline radiography, (C) Restorative procedure, (D) Immediately after the restorative procedure, (E) clinical evaluation after 24-month follow-up, restorations rated as “clinically very good” in all categories of the FDI criteria. (F) Tooth 46 with an amalgam restoration to be replaced, (G) tooth 46 immediately after the restorative procedure, (H) Clinical evaluation after 24-month follow-up, where it is observed the composite restoration with marginal staining, rated as “clinically good” according to the FDI criteria. (I) Teeth 14 and 15 with amalgam restorations to be replaced, (J) immediately after the restorative procedure, (K) clinical evaluation after 24-month follow-up, where it is possible to observe minimal discrepancies of marginal adaptation in the proximal areas of both restorations (14 distal, 15 mesial), as well as a minimal color mismatch, both rated as “clinically good” according to the FDI criteria

The restorative procedures were implemented exactly as planned, and no modification was conducted. All baseline details regarding the research subjects and characteristics of the restored cavities were described in Table 1 . Figure 3 shows representative photographs of the clinical procedures and follow-ups. The overall Cohen kappa statistics showed an excellent agreement of inter- (0.86) and intra-examiners (0.75) during the follow-up recalls.

### Functional properties

#### Fracture and retention

Table 2 shows all data regarding the follow-up times. However, only the 24-month data are described here. One restoration for SETIn showed fractures classified as clinically unsatisfactory but repairable, and three restorations (1 for SEEBSy and 2 for SEEIn) showed fractures classified as clinically poor. According to the FDI criteria, the 24-month fracture and retention rates (95%CI) were 97.8% (95%CI 88.9 - 99.6) for SEEBSy, 100% (95%CI 92.3 - 100.0) for SETBSy, 100% (95%CI 91.6 - 100.0) for SEEBCa, 100% (95%CI 91.4 - 100.0) for SETBCa, 95.2% (95%CI 84.5 - 98.7) for SEEIn, and 97.7% (95%CI 87.8 - 99.6) for SETIn ( Table 3 ).

**Table 2:** Number of evaluated restorations for each experimental group (*) classified according to the World Dental Federation (FDI) criteria. ^27^

FDI Criteria		(**)	1 week					
			SEEBSy	SETBSy	SEEBCa	SETBCa	SEEIn	SETIn
Functional properties	Fracture and retention	A	52	52	47	47	48	49
		B	--	--	--	--	--	--
		C	--	--	--	--	--	--
		D	--	--	--	--	--	--
		E	--	--	--	--	--	--
	Marginal adaptation	A	52	52	47	47	48	49
		B	--	--	--	--	--	--
		C	--	--	--	--	--	--
		D	--	--	--	--	--	--
		E	--	--	--	--	--	--
	Proximal contact (***)	A	21	20	23	25	13	17
		B	--	--	--	--	--	--
		C	--	--	--	--	--	--
		D	--	--	--	--	--	--
		E	--	--	--	--	--	--
	Patient's perception	A	52	52	47	47	48	49
		B	--	--	--	--	--	--
		C	--	--	--	--	--	--
		D	--	--	--	--	--	--
		E	--	--	--	--	--	--
Esthetic properties	Marginal staining	A	52	52	47	47	48	49
		B	--	--	--	--	--	--
		C	--	--	--	--	--	--
		D	--	--	--	--	--	--
		E	--	--	--	--	--	--
	Color match	A	52	52	47	47	48	49
		B	--	--	--	--	--	--
		C	--	--	--	--	--	--
		D	--	--	--	--	--	--
		E	--	--	--	--	--	--
	Anatomic form	A	52	52	47	47	48	49
		B	--	--	--	--	--	--
		C	--	--	--	--	--	--
		D	--	--	--	--	--	--
		E	--	--	--	--	--	--
Biological properties	Post-operative (hyper-) sensitivity	A	47	50	47	46	46	47
		B	5	2	--	1	2	2
		C	--	--	--	--	--	--
		D	--	--	--	--	--	--
		E	--	--	--	--	--	--
	Recurrence of caries	A	52	52	47	47	48	49
		B	--	--	--	--	--	--
		C	--	--	--	--	--	--
		D	--	--	--	--	--	--
		E	--	--	--	--	--	--
**FDI Criteria**		(**)	**6 months**					
			**SEEBSy**	**SETBSy**	**SEEBCa**	**SETBCa**	**SEEIn**	**SETIn**
Functional properties	Fracture and retention	A	50	50	45	46	46	47
		B	--	--	2	1	1	1
		C	--	--	--	--	--	--
		D	--	--	--	--	--	--
		E	--	--	--	--	--	--
	Marginal adaptation	A	46	49	47	40	46	47
		B	4	1	--	7	1	1
		C	--	--	--	--	--	--
		D	--	--	--	--	--	--
		E	--	--	--	--	--	--
	Proximal contact (***)	A	21	20	23	25	13	17
		B	--	--	--	--	--	--
		C	--	--	--	--	--	--
		D	--	--	--	--	--	--
		E	--	--	--	--	--	--
	Patient's perception	A	50	50	47	47	47	48
		B	--	--	--	--	--	--
		C	--	--	--	--	--	--
		D	--	--	--	--	--	--
		E	--	--	--	--	--	--
Esthetic properties	Marginal staining	A	47	49	47	47	47	47
		B	3	1	--	--	--	1
		C	--	--	--	--	--	--
		D	--	--	--	--	--	--
		E	--	--	--	--	--	--
	Color match	A	50	50	47	47	47	48
		B	--	--	--	--	--	--
		C	--	--	--	--	--	--
		D	--	--	--	--	--	--
		E	--	--	--	--	--	--
	Anatomic form	A	50	50	47	47	47	48
		B	--	--	--	--	--	--
		C	--	--	--	--	--	--
		D	--	--	--	--	--	--
		E	--	--	--	--	--	--
Biological properties	Post-operative (hyper-) sensitivity	A	50	50	47	47	47	48
		B	--	--	--	--	--	--
		C	--	--	--	--	--	--
		D	--	--	--	--	--	--
		E	--	--	--	--	--	--
	Recurrence of caries	A	50	50	47	47	47	48
		B	--	--	--	--	--	--
		C	--	--	--	--	--	--
		D	--	--	--	--	--	--
		E	--	--	--	--	--	--
**FDI Criteria**		(**)	**12 months**					
			**SEEBSy**	**SETBSy**	**SEEBCa**	**SETBCa**	**SEEIn**	**SETIn**
Functional properties	Fracture and retention	A	50	49	44	43	42	45
		B	--	1	2	1	3	1
		C	--	--	--	--	--	--
		D	--	--	--	--	--	1
		E	--	--	--	1	1	--
	Marginal adaptation	A	42	47	46	37	42	41
		B	8	3	--	7	3	5
		C	--	--	--	--	--	--
		D	--	--	--	--	--	--
		E	--	--	--	--	--	--
	Proximal contact (***)	A	20	20	22	25	13	16
		B	--	--	--	--	--	--
		C	--	--	--	--	--	--
		D	--	--	--	--	--	--
		E	--	--	--	--	--	--
	Patient's perception	A	50	50	46	44	45	46
		B	--	--	--	--	--	--
		C	--	--	--	--	--	--
		D	--	--	--	--	--	--
		E	--	--	--	--	--	--
Esthetic properties	Marginal staining	A	49	49	46	44	39	40
		B	1	1	--	--	6	6
		C	--	--	--	--	--	--
		D	--	--	--	--	--	--
		E	--	--	--	--	--	--
	Color match	A	50	50	46	44	45	46
		B	--	--	--	--	--	--
		C	--	--	--	--	--	--
		D	--	--	--	--	--	--
		E	--	--	--	--	--	--
	Anatomic form	A	50	50	46	44	45	46
		B	--	--	--	--	--	--
		C	--	--	--	--	--	--
		D	--	--	--	--	--	--
		E	--	--	--	--	--	--
Biological properties	Post-operative (hyper-) sensitivity	A	50	50	46	44	44	46
		B	--	--	--	--	--	--
		C	--	--	--	--	--	--
		D	--	--	--	--	--	--
		E	--	--	--	--	1	--
	Recurrence of caries	A	50	50	46	44	45	46
		B	--	--	--	--	--	--
		C	--	--	--	--	--	--
		D	--	--	--	--	--	--
		E	--	--	--	--	--	--
**FDI Criteria**		(**)	**24 months**					
			**SEEBSy**	**SETBSy**	**SEEBCa**	**SETBCa**	**SEEIn**	**SETIn**
Functional properties	Fracture and retention	A	46	46	41	40	39	42
		B	--	--	1	1	2	--
		C	--	--	--	--	--	--
		D	--	--	--	--	--	1
		E	1	--	--	--	2	--
	Marginal adaptation	A	40	35	42	34	29	29
		B	6	11	--	7	12	13
		C	--	--	--	--	--	--
		D	--	--	--	--	--	--
		E	--	--	--	--	--	--
	Proximal contact (***)	A	18	19	22	25	11	16
		B	--	--	--	--	--	--
		C	--	--	--	--	--	--
		D	--	--	--	--	--	--
		E	--	--	--	--	--	--
	Patient's perception	A	45	46	42	41	41	42
		B	1	--	--	--	--	--
		C	--	--	--	--	--	--
		D	--	--	--	--	--	--
		E	--	--	--	--	--	--
Esthetic properties	Marginal staining	A	44	44	41	40	34	37
		B	2	2	1	1	7	5
		C	--	--	--	--	--	--
		D	--	--	--	--	--	--
		E	--	--	--	--	--	--
	Color match	A	41	42	42	41	36	37
		B	5	4	--	--	5	5
		C	--	--	--	--	--	--
		D	--	--	--	--	--	--
		E	--	--	--	--	--	--
	Anatomic form	A	45	46	42	41	41	42
		B	1	--	--	--	--	--
		C	--	--	--	--	--	--
		D	--	--	--	--	--	--
		E	--	--	--	--	--	--
Biological properties	Post-operative (hyper-) sensitivity	A	46	46	42	41	41	42
		B	--	--	--	--	--	--
		C	--	--	--	--	--	--
		D	--	--	--	--	--	--
		E	--	--	--	--	--	--
	Recurrence of caries	A	46	46	42	41	40	42
		B	--	--	--	--	--	--
		C	--	--	--	--	--	--
		D	--	--	--	--	--	--
		E	--	--	--	--	1	--

(*) SEEBSy, self-etch with selective enamel etching and bulk in syringe; SETBSy, self-etch and bulk in syringe; SEEBCa, self-etch with selective enamel etching and bulk in capsule; SETBCa, self-etch and bulk in capsule; SEEIn, self-etch with selective enamel etching and incremental composite; SETIn, self-etch and incremental composite.

(**) A = Clinically very good; B = Clinically good; C = Clinically sufficient/satisfactory; D = Clinically unsatisfactory; E = Clinically poor.

(***) Only for class II restorations.

**Table 3:** Absolut risk (95%CI) and relative risk (95%CI) for outcome fracture and retention for different research groups after 24 months of clinical evaluation

	Absolute risk (95%CI)	Relative risk (95%CI)*
SEEBSy	97.8 (88.9–99.6)	0.86 (0.37–1.97)
SETBSy	100 (92.3–100.0)	0.86 (0.37–1.97)
SEEBCa	100 (91.6–100.0)	0.57 (0.21–1.51)
SETBCa	100 (91.4–100.0)	0.85 (0.36–2.01)
SEEIn	95.2 (84.5–98.7)	0.98 (0.48–1.98)
SETIn	97.7 (87.8–99.6)	_

* Related to group SETIn

**Figure 4:**
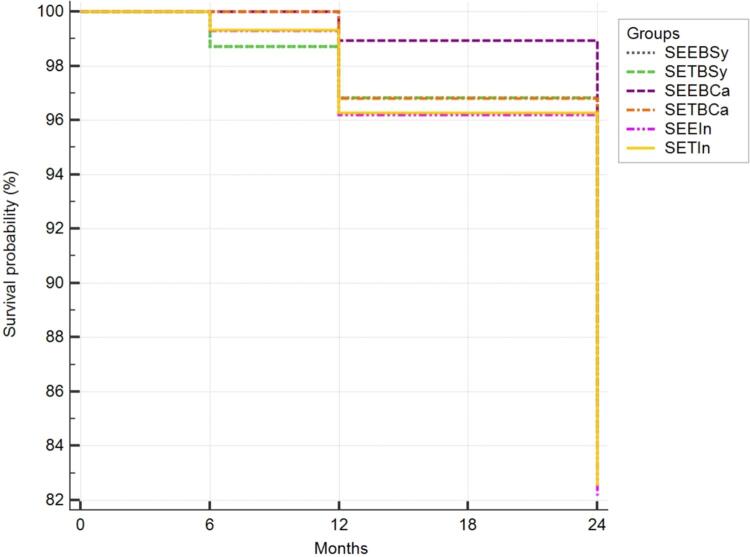
Survival curves for all groups. Abbreviations: SEEBSy, self-etch with selective enamel etching and bulk in syringe; SETBSy, self-etch and bulk in syringe; SEEBCa, self-etch with selective enamel etching and bulk in capsule; SETBCa, self-etch and bulk in capsule; SEEIn, self-etch with selective enamel etching and incremental composite; SETIn, self-etch and incremental composite

The Kaplan-Meier curves did not show any significant differences (Logrank test, p=0.88) among the cumulative probability of the primary endpoint, which was fracture and retention ( Figure 4 ). Table 4 depicts the paired comparisons among the six research groups as the hazard ratios. The fact that the 95%CI interval of the hazard ratio crosses the null value of 1 means that none of the paired groups showed any significant difference.

**Table 4:** Fracture and retention loss hazard ratio (95%CI) for pairwise comparison of different groups

Pairwise comparison	Hazard ratio (95%CI)
SEEBSy vs. SETIn	0.86 (0.35–2.08)
SETBSy vs. SETIn	0.86 (0.35–2.08)
SEEBCa vs. SETIn	0.57 (0.23–1.41)
SETBCa vs. SETIn	0.85 (0.34–2.12)
SEEIn vs. SETIn	1.02 (0.41–2.52)
SEEBSy vs. SETBSy	1.00 (0.42–2.40)
SEEBCa vs. SETBCa	0.67 (0.27–1.67)

Regarding the type of cavity (class I and II), the loss of restorations was distributed according to the following: for SEEBSy (class I: 50%, class II: 50%), for SETBSy (class I: 45%, class II: 55%), for SETBCa (class I: 43%, class II: 57%), for SEEIn (class I: 50%, class II: 50%), and for SETIn (class I: 47%, class II: 53%). Regarding the treatment reason (carious lesion and restoration replacement), the loss of restorations was distributed as follows: for SEEBSy (carious lesion: 34%, restoration replacement: 66%), for SETBSy (carious lesion: 55%, restoration replacement: 45%), for SETBCa (carious lesion: 57%, restoration replacement: 43%), for SEEIn (carious lesion: 42%, restoration replacement: 58%), and for SETIn (carious lesion: 53%, restoration replacement: 47%). Although no statistical analysis was conducted, mainly due to the low number of lost restorations, the descriptive statistics suggested that a similar percentage of restoration was lost, regardless of the type of cavity and treatment reason.

#### Marginal adaptation

At the 24-month recall, 49 restorations (six for SEEBSy; 11 for SETBSy; seven for SETBCa; 12 for SEEIn; and 13 for SETIn) showed minor marginal adaptation discrepancies according to the FDI criteria ( Table 2 ). When restorations made with SEE and SET were compared, significantly fewer marginal discrepancies were observed in the selective enamel etching strategy compared to the self-etch strategy (p=0.003; Table 2 ). When the different composites were compared, a significantly smaller number of restorations with marginal discrepancies were observed in restorations performed with bulk-fill in capsules and bulk-fill in syringes, compared to restorations performed with the incremental technique (p=0.003; Table 2 ). No significant difference was detected in any other comparison of groups at the 6- and 12-month recalls (p>0.05; Table 2 ). All marginal adaptations observed in this study were in the enamel margins.

#### Proximal contact quality

No restoration showed any problem regarding proximal contact according to the FDI criteria after six, 12, and 24 months of clinical evaluation (p=1.00; Table 2 ).

#### Patient’s perception

Only one restoration for SEEIn presented minor problems regarding the patient perception at the 24-month follow-up according to the FDI criteria ( Table 2 ). No significant difference was detected between any pair of groups at the 24-month follow-ups (p=1.00; Table 2 ).

## Esthetic properties

### Marginal staining

After 24 months of clinical evaluation, 18 restorations (two for SEEBSy; two for SETBSy; one for SEEBCa; and one for SETBCa, seven for SEEIn; and five for SETIn) showed minor marginal staining according to the FDI criteria ( Table 2 ). Regarding restorations made with SEE or SET, no significant difference was found between groups at 24-month recall (p>0.05; Table 2 ). When different composites were compared, a significant and smaller number of restorations with marginal staining was observed in restorations performed with bulk-fill in capsules and in syringes when compared to restorations performed with the incremental technique after a 24-month recall (p=0.002). No significant difference was detected in any other comparison at the 6- and 12-month recalls (p>0.05; Table 2 ).

### Color match

At the 24-month follow-up, 19 restorations showed minor discrepancies in the color match (five for SEEBSy; four for SETBSy; five for SEEIn; and five for SETIn) according to the FDI criteria ( Table 2 ). Restorations made with SEE or SET showed no significant difference between groups at 24-month recall (p>0.05; Table 2 ). Restorations made with different composites showed significant differences: a smaller number of restorations with color mismatch were observed in restorations performed with bulk-fill in capsules (SEEBCa and SETBCa) compared to other groups after 24-month recall (p=0.002; Table 2 ). No significant difference was detected in any other comparison of groups at the 6- and 12-month recalls (p>0.05; Table 2 ).

### Anatomic form

At the 24-month follow-up, one restoration for SEEBSy presented minor anatomic form problems according to the FDI criteria ( Table 2 ). No significant difference was detected between any pair of groups at the 6-, 12-, and 24-month follow-ups (p=1.00; Table 2 ).

## Biological properties

### Post-operative (hyper-) sensitivity

One restoration for SEEIn presented unacceptable post-operative sensitivity after 12 months according to the FDI criteria ( Table 2 ) and had to be replaced. The patient did not return to the 24-month recall. None of the remaining restorations showed post-operative sensitivity after 24 months of clinical evaluation ( Table 2 ). No significant difference was detected between any pair of groups at 24-month follow-ups (p=1.00; Table 2 ).

### Recurrence of caries

No restorations had a recurrence of caries at the 1-week evaluation or at the 6, or 12-month recall according to the FDI criteria (p=1.00; Table 2 ). One restoration for SEEIn showed a recurrence of caries after 24 months according to the FDI criteria (p=0.3812; Table 2 ) and had to be replaced after this period.

## Discussion

In this study, some clinical parameters of the performance of restorations were influenced by the bulk-fill composite packaging and SEE. After 24 months of clinical use, restorations placed using the bulk-fill composite, mainly in capsules, showed better clinical performance than those placed with the incremental technique. Furthermore, restorations placed with SEE followed by adhesive application showed better performance than those placed using only the SET strategy.

Initially, considering the parameters of the FDI criteria for evaluating restorative materials’ functional properties, the main outcome from the point of view of longitudinal evaluation was the restoration fracture and retention. This study showed a few cases of fracture in various groups after 24 months, with no significant difference between them, regardless of the packaging of composite resin (syringe vs. capsule) and the adhesive strategy (SEE vs. SET). Therefore, the first and second null hypotheses were accepted. The reason for fractures of composite restorations in this study could be non-diagnosed parafunctional habits or changes in some patients’ behavior during the follow-up times and should be considered in addition to the restorative material itself. However, the similar satisfactory clinical performance of bulk-fill restorations and those with incremental resin agrees with previous literature.^8 , 12 , 30 - 33^

The studied composites’ low fracture rate, as well as the satisfactory anatomic form and proximal contact, could be explained by their larger amount of filler. Moreover, the materials contain additional zirconia filler, and zirconia/silica fillers (2.5 and 5.0 wt%) replace those of glass, improving some mechanical properties such as flexural strength and fracture resistance.^34^ Bulk-fill materials present an increased translucency compared to incremental composite resins,^35^ which allows for a sufficient depth of cure^36^ by reducing light scattering and improving deeper blue-light penetration,^37^ thus enhancing these material’s mechanical properties.^1^

In our study, this translucency characteristic of bulk-fill composite resins seems not to affect the restorations’ color match after a 24-month evaluation. Even so, a larger percentage of restorations with color mismatch was observed in the incremental groups (12%) and bulk-fill composite resin in syringe groups (9%) than in the bulk-fill composite resin in capsules (0%). This behavior could be related to several factors. First, composites have different chemical compositions because only the Filtek Supreme Ultra composite resin, which was used in the incremental group, contains the hydrophilic monomer triethyleneglycol dimethacrylate, which makes the composite more prone to incorporate pigments and staining over time due to its higher water absorption capacity.^38 , 39^ Second, the bulk-fill composites have different opacities. According to the manufacturer, Filtek One Bulk-Fill resin in capsules is slightly opaquer than Filtek Bulk-Fill Posterior Restorative resin in syringes and therefore seems to produce a better color match in the former. However, *in vitro* studies evaluating these colorimetric differences should be conducted to provide stronger evidence.

The marginal staining and marginal adaptation were the categories in which bulk-fill composite resin restorations showed better results compared to those made with incremental resin. Therefore, the third null hypothesis was partially rejected. When the composition of Filtek Bulk-Fill (Filtek Bulk-Fill Posterior Restorative or Filtek One Bulk-Fill) was evaluated, some components such as an aromatic dimethacrylate (AUDMA), additional fragmentation molecules (AFM), urethane dimethacrylate (UDMA), and 1,12-dodecane dimethacrylate (DDMA) were found in its resin matrix.^40^ The inclusion of these monomers into the resin matrix allows the polymeric network to relax, providing a potential mechanism for stress relief, which enables the network to reorganize, thus decreasing the polymerization shrinkage stress generated.^41^ This stress relief promotes fewer marginal alterations and consequently decreases the risk of marginal staining compared to incremental resins.^42^ However, controversial results are observed in the literature regarding these issues. For instance, although some authors observed superior results in the groups with bulk-fill resins,^8^ others^9 - 12^ did not observe differences between bulk-fill resins and incremental resins in these clinical parameters. Comparing their results with ours is difficult, mainly because of the different evaluation criteria used. While we used the FDI criteria, other studies^9 , 11^ used the modified United States Public Health Service (USPHS).^43^ It is a consensus that the FDI criteria are more sensitive and precise in detecting minor failures than the modified USPHS criteria.^15 , 44^ This means that, while in our study early failures are already being detected, in these other studies,^9 , 11^ these same failures occurred but were not observable.

However, the most interesting results related to marginal adaptation were observed in the evaluation of the commercial presentation of bulk-fill composite resins. Composites in capsules showed better marginal adaptation after a 24-month recall than composites in syringes. Several factors may be involved in the effective marginal adaptation of a resin composite to the cavity.^45^ However, we hypothesize that the better marginal adaptation observed in our study for bulk-fill composite used in capsules rather than in syringes could occur due to the direct material application with the aid of a Centrix syringe or dispenser that facilitates the insertion of the composite resin into the cavity without the use of spatulas. Consequently, this procedure may result in a reduced probability of the presence of voids and porosities in the final restoration.^45 , 46^ Similar results were also observed in non-carious cervical restorations.^17 , 47^ Additionally, the use of bulk-fill in capsules required less handling than bulk-fill in syringes, which could somehow clinically favor the restorative procedure. This easier manipulation of bulk-fill in capsules was evidenced by the shorter clinical time required to restore them compared to bulk-fill in syringes, in the first part of this study.^5^ Unfortunately, to the authors’ knowledge, no previous studies have been conducted to test this hypothesis; therefore, more clinical studies comparing materials presented in syringes or capsules should be conducted to corroborate our observations.

Regarding the secondary outcomes of the adhesive strategy, our study showed a difference only in the marginal adaptation category, whereas the universal adhesive applied with SEE showed better marginal adaptation than the SET mode. Therefore, the fourth null hypothesis was partially rejected. Studies frequently report that the universal adhesives applied in the SET mode did not properly etch the enamel surface.^48^ This behavior was also observed in the larger discrepancies in marginal adaptation at the enamel margins in the SET compared to the SEE mode in non-carious cervical lesions.^17 , 47^ Although this study showed marginal defects in restorations, they seem not to impact the post-operative sensitivity or the development of new carious lesions at the margin of restorations after 24 months of follow-ups, which also agrees with the previous studies.^10 , 49^

It was reported that some baseline characteristics of cavities could affect the survival of posterior restorations, such as cavity extensions (class I or II).^50^ In fact, a recent study showed that “larger cavities” statistically suffered more failure than “small cavities,” regardless of the material used.^51^ On the other hand, we found a similar percentage of restoration loss in each type of cavity (class I: 47%, class II: 53%), showing that this effect of the baseline characteristic on the fracture and retention rate in the materials seems irrelevant, at least in our study. Together with the treatment reason (carious lesion or restoration replacement), this showed a similar percentage of restoration loss between them (carious lesion: 49%, restoration replacement: 51%), which seems an unimportant factor in the restoration longevity. However, a regression statistical analysis should be conducted in future studies to provide further evidence of the influence of those baseline characteristics’ effect on restoration clinical performance.

One of the limitations of this study is that although some significant differences were observed between the groups after 24 months, which is a medium-term clinical follow-up, the defects observed did not impact the patients’ perception and could be easily resolved by repolishing the restorations.^27^ However, future studies with longer follow-ups need to be conducted to determine whether this difference can be confirmed. Another limitation is that the number of restorations per patient showed some variability (three or more by each patient) due to the difficulty of recruiting patients with six restorations each, which is an ideal condition for this study. Although this is a common situation in the dental literature,^8^ it may have caused a clustering effect, whose impact on the results was not considered and should be considered in future studies. However, 6% of all restorations in our study were inserted in patients with less than four teeth to be restored, which may have had a small impact on the present study’s overall results.

Finally, the characteristics of the participants included in our study were good general health and acceptable oral hygiene, which could result in participants with low caries risk; therefore, a caries risk assessment was not conducted. However, a systematic analysis of participants’ caries risk assessment would be important to identify patient factors’ effect on restorations’ clinical performance,^52^ mainly if restorations performed in high-caries-risk patients showed a higher failure rate than those in low-risk patients.^53^ Clinical studies should be conducted to evaluate bulk-fill composite restorations’ performance, including the caries risk assessment to compare high- and low-risk patients.

## Conclusion

After 24 months of clinical service, all materials under investigation exhibited satisfactory restoration qualities.

Class I and II restorations performed with bulk-fill composites showed similar survival rates regardless of the composite packaging and the use or non-use of SEE.

Restorations performed with bulk-fill composite in syringes presented fewer marginal defects than the composite applied with the incremental technique but more than those made with bulk-fill composite in capsules.

Restorations performed with SEE presented a better marginal adaptation than those with the SET strategy.

## References

[1] 1 - Van Ende A, De Munck J, Lise DP, Van Meerbeek B. Bulk-fill composites: a review of the current literature. J Adhes Dent. 2017;19(2):95-109. doi: 10.3290/j.jad.a38141 10.3290/j.jad.a38141 28443833

[2] 2 - Ferracane JL, Lawson NC. Probing the hierarchy of evidence to identify the best strategy for placing class II dental composite restorations using current materials. J Esthet Restor Dent. 2021;33(1):39-50. doi: 10.1111/jerd.12686 10.1111/jerd.12686 33206440

[3] 3 - Ilie N, Hickel R. Investigations on a methacrylate-based flowable composite based on the SDR™ technology. Dent Mater. 2011;27(4):348-55. doi: 10.1016/j.dental.2010.11.014 10.1016/j.dental.2010.11.014 21194743

[4] 4 - El-Safty S, Silikas N, Watts DC. Creep deformation of restorative resin-composites intended for bulk-fill placement. Dent Mater. 2012;28(8):928-35. doi: 10.1016/j.dental.2012.04.038 10.1016/j.dental.2012.04.038 22656273

[5] 5 - Tardem C, Albuquerque EG, Lopes LS, Marins SS, Calazans FS, Poubel LA, et al. Clinical time and postoperative sensitivity after use of bulk-fill (syringe and capsule) vs. incremental filling composites: a randomized clinical trial. Braz Oral Res. 2019;33(0):e089. doi: 10.1590/1807-3107bor-2019.vol33.0089 10.1590/1807-3107bor-2019.vol33.0089 31531552

[6] 6 - van Dijken JWV, Pallesen U. Bulk-filled posterior resin restorations based on stress-decreasing resin technology: a randomized, controlled 6-year evaluation. Eur J Oral Sci. 2017;125(4):303-9. doi: 10.1111/eos.12351 10.1111/eos.12351 28524243

[7] 7 - Veloso SR, Lemos CA, Moraes SL, Vasconcelos BC, Pellizzer EP, Monteiro GQ. Clinical performance of bulk-fill and conventional resin composite restorations in posterior teeth: a systematic review and meta-analysis. Clin Oral Investig. 2019;23(1):221-33. doi: 10.1007/s00784-018-2429-7 10.1007/s00784-018-2429-7 29594349

[8] 8 - Yazici AR, Antonson SA, Kutuk ZB, Ergin E. Thirty-six-month clinical comparison of bulk fill and nanofill composite restorations. Oper Dent. 2017;42(5):478-85. doi: 10.2341/16-220-C 10.2341/16-220-C 28581919

[9] 9 - Atabek D, Aktaş N, Sakaryali D, Bani M. Two-year clinical performance of sonic-resin placement system in posterior restorations. Quintessence Int. 2017;48(9):743-51. doi: 10.3290/j.qi.a38855 10.3290/j.qi.a38855 28849804

[10] 10 - Loguercio AD, Rezende M, Gutierrez MF, Costa TF, Armas-Vega A, Reis A. Randomized 36-month follow-up of posterior bulk-filled resin composite restorations. J Dent. 2019;85:93-102. doi: 10.1016/j.jdent.2019.05.018 10.1016/j.jdent.2019.05.018 31100332

[11] 11 - Balkaya H, Arslan S. A Two-year clinical comparison of three different restorative materials in Class II cavities. Oper Dent. 2020;45(1):E32-42. doi: 10.2341/19-078-C 10.2341/19-078-C 31738696

[12] 12 - Torres CR, Jurema AL, Souza MY, Di Nicoló R, Borges AB. Bulk-fill versus layering pure ormocer posterior restorations: a randomized split-mouth clinical trial. Am J Dent. 2021;34(3):143-9. 34143584

[13] 13 - Kaisarly D, ElGezawi M, Haridy R, Elembaby A, Aldegheishem A, Alsheikh R, et al. Reliability of Class II bulk-fill composite restorations with and without veneering: a two-year randomized clinical control study. Oper Dent. 2021;46(5):491-504. doi: 10.2341/19-290-c 10.2341/19-290-C 35486510

[14] 14 - Loguercio AD, Paula EA, Hass V, Luque-Martinez I, Reis A, Perdigão J. A new universal simplified adhesive: 36-month randomized double-blind clinical trial. J Dent. 2015;43(9):1083-92. doi: 10.1016/j.jdent.2015.07.005 10.1016/j.jdent.2015.07.005 26159382

[15] 15 - Matos TP, Perdigão J, Paula E, Coppla F, Hass V, Scheffer RF, et al. Five-year clinical evaluation of a universal adhesive: a randomized double-blind trial. Dent Mater. 2020;36(11):1474-85. doi: 10.1016/j.dental.2020.08.007 10.1016/j.dental.2020.08.007 32933775

[16] 16 - Zanatta RF, Silva TM, Esper M, Bresciani E, Gonçalves S, Caneppele T. Bonding performance of simplified adhesive systems in noncarious cervical lesions at 2-year follow-up: a double-blind randomized clinical trial. Oper Dent. 2019;44(5):476-87. doi: 10.2341/18-049-C 10.2341/18-049-C 30702405

[17] 17 - Albuquerque EG, Warol F, Calazans FS, Poubel LA, Marins SS, Matos T, et al. A new dual-cure universal simplified adhesive: 18-month randomized multicenter clinical trial. Oper Dent. 2020;45(5):E255-70. doi: 10.2341/19-144-C 10.2341/19-144-C 33170938

[18] 18 - van Dijken JW, Pallesen U. Three-year randomized clinical study of a one-step universal adhesive and a two-step self-etch adhesive in Class II composite restorations. J Adhes Dent. 2017;19(4):287-94. doi: 10.3290/j.jad.a38867 10.3290/j.jad.a38867 28849796

[19] 19 - Çakır NN, Demirbuga S. The effect of five different universal adhesives on the clinical success of class I restorations: 24-month clinical follow-up. Clin Oral Investig. 2019;23(6):2767-76. doi: 10.1007/s00784-018-2708-3 10.1007/s00784-018-2708-3 30368662

[20] 20 - Schulz KF, Altman DG, Moher D. CONSORT 2010 statement: updated guidelines for reporting parallel group randomised trials. Int J Surg. 2011;9(8):672-7. doi: 10.1016/j.ijsu.2011.09.004 10.1016/j.ijsu.2011.09.004 22019563

[21] 21 - Costa TR, Loguercio AD, Reis A. Effect of enamel bevel on the clinical performance of resin composite restorations placed in non-carious cervical lesions. J Esthet Restor Dent. 2013;25(5):346-56. doi: 10.1111/jerd.12042 10.1111/jerd.12042 24148985

[22] 22 - Greene JC, Vermillion JR. The simplified oral hygiene index. J Am Dent Assoc. 1964;68:7-13. doi: 10.14219/jada.archive.1964.0034 10.14219/jada.archive.1964.0034 14076341

[23] 23 - Hickel R, Brüshaver K, Ilie N. Repair of restorations: criteria for decision making and clinical recommendations. Dent Mater. 2013;29(1):28-50. doi: 10.1016/j.dental.2012.07.006 10.1016/j.dental.2012.07.006 22867859

[24] 24 - Papapanou PN, Sanz M, Buduneli N, Dietrich T, Feres M, Fine DH, et al. Periodontitis: consensus report of workgroup 2 of the 2017 World Workshop on the Classification of Periodontal and Peri-Implant Diseases and Conditions. J Periodontol. 2018;89 Suppl 1:S173-82. doi: 10.1002/jper.17-0721 10.1002/JPER.17-0721 29926951

[25] 25 - Young DA, Nový BB, Zeller GG, Hale R, Hart TC, Truelove EL. The American Dental Association Caries Classification System for clinical practice: a report of the American Dental Association Council on Scientific Affairs. J Am Dent Assoc. 2015;146(2):79-86. doi: 10.1016/j.adaj.2014.11.018 10.1016/j.adaj.2014.11.018 25637205

[26] 26 - van Dijken JW, Pallesen U. A randomized controlled three year evaluation of “bulk-filled” posterior resin restorations based on stress decreasing resin technology. Dent Mater. 2014;30(9):e245-51. doi: 10.1016/j.dental.2014.05.028 10.1016/j.dental.2014.05.028 24958689

[27] 27 - Hickel R, Peschke A, Tyas M, Mjör I, Bayne S, Peters M, et al. FDI World Dental Federation: clinical criteria for the evaluation of direct and indirect restorations. Update and clinical examples. J Adhes Dent. 2010;12(4):259-72. doi: 10.3290/j.jad.a19262 10.3290/j.jad.a19262 20847997

[28] 28 - Hickel R, Roulet JF, Bayne S, Heintze SD, Mjör IA, Peters M, et al. Recommendations for conducting controlled clinical studies of dental restorative materials. Science Committee Project 2/98--FDI World Dental Federation study design (Part I) and criteria for evaluation (Part II) of direct and indirect restorations including onlays and partial crowns. J Adhes Dent. 2007;9 Suppl 1:121-47. doi: 10.3290/j.jad.a11976 18341239

[29] 29 - Spineli LM, Fleming PS, Pandis N. Addressing missing participant outcome data in dental clinical trials. J Dent. 2015;43(6):605-18. doi: 10.1016/j.jdent.2015.03.007 10.1016/j.jdent.2015.03.007 25837533

[30] 30 - Maran BM, Geus JL, Gutiérrez MF, Heintze S, Tardem C, Barceleiro MO, et al. Nanofilled/nanohybrid and hybrid resin-based composite in patients with direct restorations in posterior teeth: a systematic review and meta-analysis. J Dent. 2020;99:103407. doi: 10.1016/j.jdent.2020.103407 10.1016/j.jdent.2020.103407 32526348

[31] 31 - Durão MA, Andrade AK, Prado A, Veloso SR, Maciel LM, Montes M, et al. Thirty-six-month clinical evaluation of posterior high-viscosity bulk-fill resin composite restorations in a high caries incidence population: interim results of a randomized clinical trial. Clin Oral Investig. 2021;25(11):6219-37. doi: 10.1007/s00784-021-03921-9 10.1007/s00784-021-03921-9 33821322

[32] 32 - Kunz PV, Wambier LM, Kaizer MD, Correr GM, Reis A, Gonzaga CC. Is the clinical performance of composite resin restorations in posterior teeth similar if restored with incremental or bulk-filling techniques? A systematic review and meta-analysis. Clin Oral Investig. 2022;26(3):2281-97. doi: 10.1007/s00784-021-04337-1 10.1007/s00784-021-04337-1 35031879

[33] 33 - Abreu NM, Sousa FB, Dantas RV, Leite PK, Batista AU, Montenegro RV. Longevity of bulk fill and ormocer composites in permanent posterior teeth: systematic review and meta-analysis. Am J Dent. 2022;35(2):89-96. 35506964

[34] 34 - Ikeda I, Otsuki M, Sadr A, Nomura T, Kishikawa R, Tagami J. Effect of filler content of flowable composites on resin-cavity interface. Dent Mater J. 2009;28(6):679-85. doi: 10.4012/dmj.28.679 10.4012/dmj.28.679 20019418

[35] 35 - Grazioli G, Cuevas-Suárez CE, Nakanishi L, Francia A, Moraes RR. Physicochemical characterization of two bulk fill composites at different depths. Restor Dent Endod. 2021;46(3):e39. doi: 10.5395/rde.2021.46.e39 10.5395/rde.2021.46.e39 PMC8411009 34513645

[36] 36 - Makhdoom SN, Campbell KM, Carvalho RM, Manso AP. Effects of curing modes on depth of cure and microtensile bond strength of bulk fill composites to dentin. J Appl Oral Sci. 2020;28:e20190753. doi: 10.1590/1678-7757-2019-0753 10.1590/1678-7757-2019-0753 PMC7340207 32638829

[37] 37 - Ilie N, Keßler A, Durner J. Influence of various irradiation processes on the mechanical properties and polymerisation kinetics of bulk-fill resin based composites. J Dent. 2013;41(8):695-702. doi: 10.1016/j.jdent.2013.05.008 10.1016/j.jdent.2013.05.008 23707645

[38] 38 - Backes CN, França FM, Turssi CP, Amaral F, Basting RT. Color stability of a bulk-fill composite resin light-cured at different distances. Braz Oral Res. 2020;34:e119. doi: 10.1590/1807-3107bor-2020.vol34.0119 10.1590/1807-3107bor-2020.vol34.0119 33146315

[39] 39 - Ferracane JL. Hygroscopic and hydrolytic effects in dental polymer networks. Dent Mater. 2006;22(3):211-22. doi: 10.1016/j.dental.2005.05.005 10.1016/j.dental.2005.05.005 16087225

[40] 40 - Mandava J, Vegesna DP, Ravi R, Boddeda MR, Uppalapati LV, Ghazanfaruddin MD. Microtensile bond strength of bulk-fill restorative composites to dentin. J Clin Exp Dent. 2017;9(8):e1023-8. doi: 10.4317/jced.53965 10.4317/jced.53965 PMC5601103 28936294

[41] 41 - Falsafi A, Oxman JD, Tse PH, Ton TT. Longer-term postcure measurement of cuspal deformation induced by dimensional changes in dental materials. Int J Dent. 2015;2015:915071. doi: 10.1155/2015/915071 10.1155/2015/915071 PMC4519530 26257783

[42] 42 - Rizzante FA, Mondelli RF, Furuse AY, Borges AF, Mendonça G, Ishikiriama SK. Shrinkage stress and elastic modulus assessment of bulk-fill composites. J Appl Oral Sci. 2019;27:e20180132. doi: 10.1590/1678-7757-2018-0132 10.1590/1678-7757-2018-0132 PMC6322642 30624465

[43] 43 - Cvar JF, Ryge G. Reprint of criteria for the clinical evaluation of dental restorative materials. 1971. Clin Oral Investig. 2005;9(4):215-32. doi: 10.1007/s00784-005-0018-z 10.1007/s00784-005-0018-z 16315023

[44] 44 - Marquillier T, Doméjean S, Le Clerc J, Chemla F, Gritsch K, Maurin JC, et al. The use of FDI criteria in clinical trials on direct dental restorations: a scoping review. J Dent. 2018;68:1-9. doi: 10.1016/j.jdent.2017.10.007 10.1016/j.jdent.2017.10.007 29055692

[45] 45 - Opdam NJ, Roeters JJ, Joosten M, Veeke Ov. Porosities and voids in Class I restorations placed by six operators using a packable or syringable composite. Dent Mater. 2002;18(1):58-63. doi: 10.1016/s0109-5641(01)00020-3 10.1016/s0109-5641(01)00020-3 11740965

[46] 46 - Nie J, Wang XY, Gao XJ. [Micro-CT observations of the adaptation at gingival wall in Class II restorations with different dental restorative materials]. Beijing Da Xue Xue Bao Yi Xue Ban. 2015;47(2):317-20. Chinese. 25882952

[47] 47 - Perdigão J, Kose C, Mena-Serrano AP, Paula EA, Tay LY, Reis A, et al. A new universal simplified adhesive: 18-month clinical evaluation. Oper Dent. 2014;39(2):113-27. doi: 10.2341/13-045-C 10.2341/13-045-C 23802645

[48] 48 - Jacker-Guhr S, Sander J, Luehrs AK. How “Universal” is adhesion? Shear bond strength of multi-mode adhesives to enamel and dentin. J Adhes Dent. 2019;21(1):87-95. doi: 10.3290/j.jad.a41974 10.3290/j.jad.a41974 30799475

[49] 49 - Reis A, Loguercio AD, Schroeder M, Luque-Martinez I, Masterson D, Cople Maia L. Does the adhesive strategy influence the post-operative sensitivity in adult patients with posterior resin composite restorations? A systematic review and meta-analysis. Dent Mater. 2015;31(9):1052-67. doi: 10.1016/j.dental.2015.06.001 10.1016/j.dental.2015.06.001 26122377

[50] 50 - Da Rosa Rodolpho PA, Donassollo TA, Cenci MS, Loguércio AD, Moraes RR, Bronkhorst EM, et al. 22-Year clinical evaluation of the performance of two posterior composites with different filler characteristics. Dent Mater. 2011;27(10):955-63. doi: 10.1016/j.dental.2011.06.001 10.1016/j.dental.2011.06.001 21762980

[51] 51 - Heck K, Manhart J, Hickel R, Diegritz C. Clinical evaluation of the bulk fill composite QuiXfil in molar class I and II cavities: 10-year results of a RCT. Dent Mater. 2018;34(6):e138-47. doi: 10.1016/j.dental.2018.03.023 10.1016/j.dental.2018.03.023 29636239

[52] 52 - Pallesen U, van Dijken JW. A randomized controlled 30 years follow up of three conventional resin composites in Class II restorations. Dent Mater. 2015;31(10):1232-44. doi: 10.1016/j.dental.2015.08.146 10.1016/j.dental.2015.08.146 26321155

[53] 53 - Opdam NJ, Bronkhorst EM, Loomans BA, Huysmans MC. 12-year survival of composite vs. amalgam restorations. J Dent Res. 2010;89(10):1063-7. doi: 10.1177/0022034510376071 10.1177/0022034510376071 20660797

